# The association between information and communication technologies, loneliness and social connectedness: A scoping review

**DOI:** 10.3389/fpsyg.2023.1063146

**Published:** 2023-03-22

**Authors:** Berkley Petersen, Najmeh Khalili-Mahani, Caitlin Murphy, Kim Sawchuk, Natalie Phillips, Karen Z. H. Li, Shannon Hebblethwaite

**Affiliations:** ^1^Laboratory for Adult Development and Cognitive Aging, Department of Psychology, Concordia University, Montreal, QC, Canada; ^2^Media Health Lab, Department of Design and Computation Arts, Milieux Institute for Arts, Culture and Technology, Concordia University, Montreal, QC, Canada; ^3^McGill Centre for Integrative Neuroscience, McGill University, Montreal, QC, Canada; ^4^Centre for Interdisciplinary Research in Rehabilitation (CRIR), Lethbridge-Layton-Mackay Rehabilitation Centre, Montreal, QC, Canada; ^5^Aging and Communication Technologies (ACT), Department of Communication Studies, Concordia University, Montreal, QC, Canada; ^6^Laboratory of Cognition, Aging and Psychophysiology (CAP), Department of Psychology, Concordia University, Montreal, QC, Canada; ^7^Department of Applied Human Sciences, Concordia University, Montreal, QC, Canada

**Keywords:** information and communication technologies, older adults, loneliness, social connectedness, wellbeing, scoping review

## Abstract

Older adults are at a higher risk of loneliness, compared to other demographics. The use of Internet Communication and Technologies (ICTs) among older adults is steadily increasing and given ICTs provide a means of enhancing social connectedness suggests they may have positive effects on reducing loneliness. Therefore, the aim of this scoping review was to examine the research that explores how ICTs may be implicated in mitigating loneliness and increasing social connectedness among older adults. After the examination of 54 articles, we identified three major themes within the literature: (1) ICTs were associated with a reduction in loneliness and increase in wellbeing. (2) ICTs promoted social connectedness by facilitating conversations. (3) Factors such as training, self-efficacy, self-esteem, autonomy, and the design/features, or affordances, of ICTs contribute toward the associations between ICT use and wellbeing. The heterogeneity of methodologies, statistical reporting, the small sample sizes of interventional and observational studies, and the diversity of the experimental contexts underline the challenges of quantitative research in this field and highlights the necessity of tailoring ICT interventions to the needs and contexts of the older users.

## 1. Introduction

### 1.1. What is known about the impact of loneliness and social isolation on older adults’ wellbeing

Later-life events, such as retirement, relocation, and death or illness among friends and family, impact both the quantity and quality of older adults’ social interactions, increasing risk of social isolation and loneliness (Ashida and Heaney, 2008). Research highlights that older adults experience a higher risk of loneliness and social isolation, as compared to other demographics (O’Rourke and Sidani, 2017; O’Rourke et al., 2018). Loneliness–the subjective feeling of lacking social resources and connections to turn to for support, companionship, and sense of security–is a consequence of social isolation (Victor et al., 2001, 2002). Evidence exists in the literature, for the serious health consequences of both loneliness (Patterson and Veenstra, 2010; Luo et al., 2012; Chopik, 2016), and social isolation (Holt-Lunstad et al., 2010; Steptoe et al., 2013; Saito et al., 2020). Among those health hazards, are increased depressive symptoms (Chopik, 2016; Santini et al., 2020), accelerated cognitive decline (Donovan et al., 2017; Griffin et al., 2020) and reduced physical activity (Schrempft et al., 2019).

The Canadian Longitudinal Study on Aging (CLSA), which contains data from a sample of Canadians aged 45–85, indicates the prevalence of loneliness (10.2%) and social isolation (5.1%) within this population (Latham-Mintus et al., 2019). Victor’s (2012) review of the prevalence of loneliness illustrates that the severity of loneliness (as measured by quantitative scores) in long term care homes is at least twice that of community-dwelling populations: 22–42% for the long-term care home population compared to 10% for the community population (Victor, 2012). A review article of 38 studies suggests that being female, non-married, older, having a poor income, a lower educational level, living alone, a low quality of social relationships, poor self-reported health, and functioning are all associated with loneliness in older adults (Cohen-Mansfield et al., 2015). There is literature that points to the need for nuance in determining how loneliness, social isolation and age are inter-related. Age is not the only cause. Data from the Statistics Canada’s General Social Survey demonstrates age is not significantly associated with loneliness when personal and social engagement characteristics are accounted for de Jong Gierveld et al. (2015).

How might we, as a society, mitigate or pre-empt the myriad of problems that are associated with loneliness and social isolation? Research indicates that increasing social engagement may be one way to instigate wellbeing and improve quality of life (Erickson and Johnson, 2011; Myhre et al., 2017). A data-driven meta-analysis by Holt-Lunstad et al. (2010) that examined 148 studies following more than 300,000 individuals over a period of 7.5 years, indicates that an individuals’ experience of social support is a significant moderator of mortality rates (increasing odds ratio by 50%), suggesting that social integration is an important predictor of survival in health interventions (Holt-Lunstad et al., 2010). A 2015 survey in the US, which recorded changes in older adults’ confidant (close contact) networks over a period of about 5 years, documents that more than 80% of participants surveyed cultivated new confidant relationships and that the growth of these confidant networks can be associated with improvements in their self-reported psychological and functional health (Cornwell and Laumann, 2015). Greater social networks with friends (in terms of size and frequency of contact) protect against depression in older adults (Singh et al., 2016), and are associated with improved wellbeing (Chen and Feeley, 2013). Additionally, individuals with higher quality social relationships defined by high level of social supports and low social strain experienced from a spouse/partner, other family members, children, or friends, seem more motivated to engage in leisure activities and reap more health benefits than those with fewer or less meaningful social relationships (Chang et al., 2014).

### 1.2. Older adults use of information and communication technologies for increasing social connections

According to Pew Research Center (2017), the use of ICT among those 65 and above has grown considerably in the past decade (Faverio, 2022). Indeed, the COVID-19 pandemic presented us with the reality that ICTs are a necessity rather than a luxury for living in digitally driven, networked societies. More than a decade ago, a meta-analysis of Internet use among older adults reported a positive association with mental health and psychosocial covariates, specifically enhanced interpersonal relationships, greater access to community resources and social inclusion (Erickson and Johnson, 2011). Various other association studies since have indicated that internet use is positively associated with active decision-making with respect to one’s health and finances, increased self-confidence, self-efficacy, and quality of life (James et al., 2013; Heo et al., 2015; Cajita et al., 2016; Khalaila and Vitman-Schorr, 2018; Silva et al., 2018). Yet, in terms of the influence of Internet use on loneliness and social isolation, the results are inconsistent.

A scoping review by Fakoya et al. (2020) identified 33 review articles describing loneliness and social isolation interventions for older adults. They reported inconsistency arising from how interventions are categorized and defined. They identified six types of ICT interventions which involved administering use or training users to interact with or learn how to use an ICT device. For example, telephone befriending, pet companions, computer and internet training, and smart technology were different types of ICTs proposed to try and improve communication and social connectedness.

The adoption of ICTs by older adults is not without its challenges. Researchers have demonstrated that a host of socioeconomic, cultural, geographical, and personal factors must be accounted for in studying older adults’ relationships with technology (Hong and Cho, 2017; Siren and Knudsen, 2017; Arcury et al., 2020; Yoon et al., 2020). Older adults often are selective in their technology use, however, social relations are important drivers for ICT use in this population (Newman et al., 2021). The use of at least one social media site among older adult Americans 65 years and older has increased from 3% in 2005 to 45% in 2021 (Pew Research Centre, 2021). Access to modern ICTs with improved and simplified user experience is steadily growing. A study of 940 residents living in 20 retirement homes in Switzerland, indicated that 21% of residents have reported using the internet, 13% have used a smartphone, and 5% have used a tablet (Seifert and Cotten, 2020). In a review of 34 studies exploring ICT use among older adults, (Khosravi et al., 2016) found that older adults interacted with at least eight types of ICTs to maintain social connections (email, video games, personal reminder information and social management systems, asynchronous peer support chat rooms, social network sites, Telecare, and 3D virtual environments). However, a similar systematic review noted that only 1 out of 25 ICT interventions effectively reduced social isolation (Ibarra et al., 2020). A qualitative study of Technologies in Later Life (TILL) in 37 rural communities indicates that older adults may welcome the introduction of ICTs to their lives by their children, and deem them useful for creating connections, while at the same time acknowledging that the adoption of ICTS do not fully correspond to their actual needs or interests (Freeman et al., 2020). Issues surrounding older adults’ adherence and acceptance of ICTs in randomized controlled trials (RCTs) has been documented by Khalili-Mahani and Sawchuk (2022). Furthermore, there remains inconsistency as to whether ICTs are efficacious in reducing loneliness and increasing social connectedness as measured with RCTs. While older adults remain at a heightened risk of loneliness and social isolation, they also demonstrate increased ICT adoption. Given the importance placed on ICTs for staying socially connected, to what extent are ICTs associated with loneliness and social connectedness in older adults? To answer this question and uncover other factors contributing to these associations we conducted a scoping review of the literature. While more recent research has focused on ICT use and wellbeing during the COVID-19 pandemic (Llorente-Barroso et al., 2021; Veiga-Seijo et al., 2021; Dhakal et al., 2022) this scoping review maps the research evidence on the associations between ICT use and loneliness and social connectedness pre-pandemic. Importantly, this review serves as a point of comparison and emphasizes the importance of in-person social contact when examining these associations, a construct that would have been omitted in the pandemic literature.

## 2. Methods

A scoping study “aims to map *rapidly* the key concepts underpinning a research area and the main sources and types of evidence available and can be undertaken as stand-alone projects in their own right, especially where an area is complex or has not been reviewed comprehensively before” (Mays et al., 2001) (pp. 194). The framework proposed by Arksey and O’Malley (2005) was selected to identify key trends in the literature regarding whether ICTs are associated with loneliness and/or social connectedness among older adults.

Importantly, in following the guidelines provided by Arksey and O’Malley (2005) we did not “address the issue of ‘synthesis,’ that is the relative weight of evidence in favor of the effectiveness of any particular intervention” or device (pp. 30). While it is important to study loneliness and social isolation in older adulthood, we chose to narrow our focus to loneliness and social connectedness. Incorporating the relation between social isolation and ICTs would have produced a much larger data set and complicated the synthesizing of our current findings.

### 2.1. Step 1: Identifying the research questions

The following research questions were developed.

The goal of our scoping review is to examine the following questions:

1. To what extent does the research literature indicate that ICTs are associated with reduced loneliness and increased social connectedness?
2. What types of ICT devices are used in the literature to examine the association among ICTs, loneliness and improved social connectedness?

### 2.2. Step 2: Identifying relevant studies

This study was conceived in the early days of the social distancing laws coming to effect in Canada (March 2020), to protect against COVID-19 contagion. We aimed to examine the existing empirical evidence to date, to guide upcoming programming and research projects that aimed to mitigate the risks of loneliness and social isolation created by the social distancing laws. In July 2020 we performed a comprehensive literature search using the electronic databases PubMed, SCOPUS, PsycINFO, Cochrane, SPORTDiscus, Academic Search Complete, and SocINDEX. The search focused on three variables: older adults, Information and Communication Technology and loneliness. The search terms were as follows: (older adults OR elderly OR seniors) AND (ICT OR digital media OR digital technology OR digital games) AND (recreation OR leisure OR social inclusion OR loneliness OR Social connectedness). Given the distinct changes in digital socialization and communication habits emerging during the COVID-19 pandemic, we have refrained to include data emerging in the past 2 years in this scoping review. The aim of the current review is thus to serve as a baseline against which emerging evidence can be compared.

### 2.3. Step 3: Study selection

**FIGURE 1:**
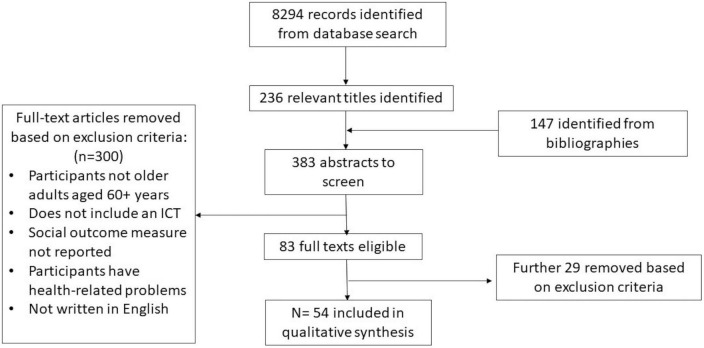
Article screening process.

The database searches yielded 8,294 records (see Figure 1). The titles were reviewed by two of the authors (BP, CM) to determine eligibility. In the case of a disagreement, a third author intervened. Titles needed to include any terms relating to older adults, a form of ICT and/or a social outcome measure. Titles were excluded if they focused on any forms of health-related problems as it is beyond the scope of this review, or if the article was part of gray literature. This reduced the number of titles to 236. We then reviewed the bibliographies of these 236 articles and included an additional 147 titles meeting the criteria from these bibliographies (total *N* = 383).

The abstracts were then screened by the same two authors using the following inclusion criteria:

• participant(s) were required to be 60 years of age or older in good general health,
• the study had to include Information and Communication Technologies.
• social outcomes (e.g., social connectedness, loneliness, social support, social engagement, inclusion, and leisure) had to be reported in the results and
• the article had to be written in English.

Exclusion criteria included:

• studies that were based solely on health-based interventions without attention to social engagement.
• studies that did not report original results for the previously mentioned criteria (e.g., Literature Reviews, Editorials and commentaries, Protocols without empirical data).
• Gray literature.

Applying the inclusion and exclusion criteria yielded 83 abstracts. To further determine if the abstracts fully met inclusion/exclusion criteria the full text articles were read with a final 54 meeting the applicable criteria and were included in the scoping review (Figure 1).

### 2.4. Step 4: Charting the data

Articles that met the selection criteria were reviewed to extract the following information into an excel spreadsheet: year of publication, geographic location, research methodology (survey, intervention, meta-analysis, qualitative research, registered clinical trials, cohort studies, and case series), sample size, sample characteristics (age, gender, social, or medical specifics), type of ICT device. See Table 1 for some characteristics of the studies.

**TABLE 1:** Study outcomes and characteristics.

References	*N*	Design	ICT type	Outcome	Age	% Female
Agmon et al., 2011	7	Case series	Digital Game- Wii Fit Exergames	Older adults enjoyed playing games with their grandchildren. The bonding time was a motivator despite the 2-month learning curve	*M* = 84, *SD* = 5, *R* = 78–92	57%
Gajadhar et al., 2010	40	Cross-sectional	Digital Game-Wood Pong	Co-located co-play was more enjoyable than virtual or mediated co-play for older adults	*M* = 68.6, *SD* = 4.7, *R* = 61–78	22.50%
Xu et al., 2016	122	Mixed quasi-experimental	Digital Game-Kinect Exergames	Reduction in social anxiety and increase in scalability for young-old participants playing with youths. Sociality improved for old-old participants playing with peers. Significant decrease in loneliness after exergaming but minimal differences across play types or age groups	Playing with elderly person: *M* = 75.91, *SD* = 6.002 playing with adolescent *M* = 76.0, *SD* = 7.43 playing alone: *M* = 73.06, *SD* = 9.38	77%
Osmanovic and Pecchioni, 2015	9	Cross-sectional-focus groups	Digital games (candy crush, Minecraft, Clash of clans, etc.)	Digital games used to stay connected to younger generations; preference for cooperative games over competitive	*M* = 64.78, *SD* = 3.46, *R* = 59–71	66.60%
Nap et al., 2009	10	Cross-sectional-focus groups	Digital games	Some participants enjoyed playing games with grandchildren, but reported no need for others to participate; played to relax or for leisure	Focus group 1: *M* = 67.8, *SD* = 3.6 focus group 2: *M* = 70.0, *SD* = 4.7	80%
Arthanat et al., 2014	12	Case series	iPad-variety	Participants reported that training increased use of iPad capabilities for leisure. Social interaction increased in proportion to importance allocated by individual	*M* = 70.6, *SD* = 6.3, *R* = 62–83	92.30%
Delello and McWhorter, 2016	19	Case series	iPad-variety	Participants reported that the iPad technology enhanced their communication with existing network	*M* = N/A, *SD* = N/A, *R* = 61–99	84.20%
Barbosa Neves et al., 2017	12	Case series	iPad-communication app	Participants reported that the App increased social interactions, but social connectedness was dependent on existing social capital	*M* = 82.5, *SD* = N/A, *R* = 74–95	66.70%
Tsai et al., 2015	21	Cross-sectional-interviews	iPad/tablet	Participants reported that Tablet use helped increase perceived connectedness with family, friends and the world.	*M* = 79.5, *SD* = N/A, *R* = 69–91	57%
Ballesteros et al., 2014	41	RCT	Prototype-AGNES–controlled social network with sensing and interaction methods to stimulate bidirectional communication	Experimental group showed significant increases in the affection dimension of wellbeing (confidence, social acceptance and satisfaction within social network) (*p* < 0.05, η^2^ = 0.34) compared to control. No change in cognitive state, depression or activities of daily living	Exp: *M* = 74, *SD* = N/A, *R* = 65–80 control: *M* = 75, *SD* = N/A, *R* = 68–85	Exp: 64.0%, control: 68.7%
Garattini et al., 2012	19	Case series	Prototype-building bridges	System provided participants opportunities to meet new people. Women were more frequent users than men. Those who reported loneliness used the system more often. Frequent users became frustrated with disengagement from others	*M* = 74, *SD* = N/A, *R* = 65–88	58%
Wherton and Prendergast, 2009	50–90 (focus groups) 5–10 (intervention)	Case series- focus groups and intervention	Prototype-building bridges	Older adults should be involved in the design of communication ICT for their age group	Age: 60+	N/A
Mitzner et al., 2019	150	RCT design but data reported only on experimental group	Proto-type-PRISM	Executive function and self-efficacy associated with continued computer use (*p* < 0.05); depression negatively correlated with mid and long-term use	*M* = 77.0, *SD* = 7.3, *R* = 65–98	79.30%
Czaja et al., 2018	300	RCT	Prototype-PRISM	Experimental group had greater increase in perceived social support (*p* < 0.004, *d* = 0.28), greater decreased loneliness (*p* < 0.04, *d* = 0.17); compared to control at 6 months, but difference gone at 12 months, potentially due to novelty. At 12 months experimental group reported greater increase in wellbeing (*p* < 0.02, *d* = 0.27).	*M* = 76.15, *SD* = 7.4, *R* = 65–98	78%
Zaine et al., 2019	4	Case series	Prototype-media parcels	Participants reported increased feelings of closeness and contact with others when using prototype	*M* = 75, *SD* = N/A, *R* = 72–82	75%
Waycott et al., 2015	N/A	Case series	Prototype-Enmesh–simplified photo and message communication	Participants reported that the prototype facilitated social engagement in home-care setting	Age: 65+	N/A
Tsai et al., 2012	52	Cohort-scenario engagement	Prototype-ShareTouch–social media platform–multimedia and games	Enrichment of social experience was dependent on self-efficacy, which was lower in the oldest-old group	*M* = 79, *SD* = N/A, *R* = 64–91	67.30%
Papa et al., 2016	40	Case series- scenario engagement	Prototype-easy reach smart TV	System perceived as useful in combatting isolation and loneliness through communicating with existing family and friends not new people	*M* = N/A, *SD* = N/A, *R* = 66–70	48%
Huang and Hsu, 2014	10	Case series	Prototype-Home TeleHealth System	Participants reported that the prototype reduced conversation gap between participant and family, encouraged family involvement in monitoring older adult health	Nine participants were over 65 years old (five participants were over 70 years). One participant was a 57-year-old male	N/A
Bobillier Chaumon et al., 2013	17	Case series	Prototype-activital software	Participants reported gains in self-esteem and bonding over ICT experience within care home, but required support and coaching	*M* = 87, *SD* = N/A, *R* = N/A	88.20%
Chi et al., 2017	10	Case series	Prototype-pet avatar	Participants reported that pet companionship was enjoyable, and they appreciated instant assistance/conversation, but conversations were superficial. Privacy, development of dependence and cost were concerns	*M* = 78.3, *SD* = N/A, *R* = 68–69	100%
Cornejo et al., 2012	1	Case study	Prototype-social media-based exergame	Participants reported the prototype facilitated bonding with younger grandchildren	*M* = 87	100%
Ballantyne et al., 2010	4	Case series	Prototype-about my age (social media platform)	Participants felt their loneliness decreased and their connectedness to the world increased	*M* = N/A, *SD* = N/A, *R* = 69–85	25%
Billipp, 2001	40	RCT	Computer-variety	Groups completing weekly computer training with either a nurse (*p* = 0.05, Effect = 0.43) or significant other (*p* = 0.03, Effect = 0.68) had improved self-esteem compared to control. Only the group with a weekly nurse computer trainer had significant change in lower levels of depression compared to control (*p* = 0.01, Effect = 0.49)	*M* = 73, *SD* = N/A, *R* = N/A Control = 10 experimental groups (*n* = 3) = 30 (breakdown not provided)	82%
Blažun et al., 2012	45	Quasi-experimental	Computer-variety	Reduction in self-reported loneliness at follow-up compared to baseline (*p* = 0.001) but over 90% of participants from both groups did not feel lonely at baseline. Significant positive correlations between email use and number of existing friends (*r* = 0.343, *p* = 0.017) and email use and number of friends made after training intervention (*r* = 0.635, *p* = 0.020)	Finland: *M* = 66.29, *SD* = 6.57, *R* = 58–80 Slovenia: *M* = 77.0, *SD* = 8.30, *R* = 58–93	Finland: 52.3% Slovenia: 66.1%
Cotten et al., 2013	205	RCT but used cross-sectional analyses on pretest data due to ongoing data collection	Computer-variety	Higher frequency of going online associated with lower levels of loneliness (*p* = 0.001) but not with lower levels of perceived social isolation (*p* = 0.06) among residents in assisted and independent living communities	*M* = 82.8, *SD* = 7.7, *R* = N/A	82.40%
Woodward et al., 2010	83	RCT	Computer-variety	Experimental group reported significantly greater self-efficacy and higher quality of life (*p* < 0.05) compared to control. No group differences in loneliness or depression	*M* = 71.85, *SD* = 7.09, *R* = 60–89	71%
Winstead et al., 2012	43	Case series	Computer-variety	Learning to use ICTs helped participants in assisted living setting overcome social barriers and connect or reconnect with others. Minimal evidence it helped overcome spatial barriers	*M* = 83.0, *SD* = 1.4, *R* = N/A	79.1%
White et al., 2016	23	Quasi-experimental	Computer-variety	Trend toward reduced loneliness in intervention group as compared to control group	Exp: *M* = 77, *SD* = 7, *R* = N/A control: *M* = 80, *SD* = 8, *R* = N/A	Exp: 84%, control: 75%
Straka and Clark, 2000	84	Case series	Computer-variety	After intervention participants felt apart of society again, it strengthened social networks. Training expanded social networks with teachers/volunteers, and participants experienced greater self-efficacy. Joy of learning mentioned by 1/3 of participants	*M* = 85.5, *SD* = N/A, *R* = 68–98	70%
Shapira et al., 2007	48	Quasi-experimental	Computer-variety	In comparison to the control group, the training group showed decreased levels of depression (*p* < 0.01, η^2^ = 0.23), and loneliness (*p* < 0.001, η^2^ = 0.51) and improvement in life satisfaction (*p* < 0.001, η^2^ = 0.55), sense of control (*p* < 0.001, η^2^ = 0.29) and life quality (*p* < 0.01, η^2^ = 0.18) at post-assessment	Exp: *M* = 80.25, *SD* = 6.50, *R* = 70–93 control: *M* = 82.60, *SD* = 5.90, *R* = 70–93	Exp: 59.1%, control: 65.4%
Pfeil et al., 2009	31	Cross-sectional- interviews	Computer-online communities	Older adults often felt uneasy when using online support communities and emails. However, online communication complemented offline communication	*M* = 69.75, *SD* = N/A, *R* = 55–91	67.70%
Nimrod and Ivan, 2019	184	Cross-sectional focus groups	Computer-general ICT	Participants reported that ICT facilitated leisure and connections, but wasted time	*M* = Varied by country, *SD* = N/A, *R* = 65–88	100%
Melenhorst et al., 2016	48	Cohort study- focus groups	Computer-email	Older adults use ICTs based on their advantages. Email and telephone were beneficial to keeping in touch over long distances	*M* = N/A, *SD* = N/A, *R* = 65–80	60.4%
Khvorostianov et al., 2011	32	Cross-sectional interviews	Computer-variety	Communicating with family and friends was main motivation for using ICT. ICTs provided virtual connection to homeland culture and leisure	*M* = 76, *SD* = N/A, *R* = 69–89	47%
Pak et al., 2020	1,698	Cross-sectional survey	Computer-variety	Web-connected ICT users were less lonely and had greater autonomy compared to non-ICT users (*p* < 0.001), and non-web ICT users (*p* < 0.001)	*R* = 80–103 no ICT *M* = 86.91, *SD* = 4.38 non-web ICT *M* = 84.73, *SD* = 3.55 web ICT: *M* = 83.92, *SD* = 3.26	No ICT: 46.3% non-web ICT 35.5% web ICT: 18.2%
Sims et al., 2017	445	Cross-sectional survey	Computer-variety	ICT use motivated by social opportunities with family. ICTs helped participants connect to friends and family more than learning new information (*p* < 0.001). Using more devices was associated with higher life satisfaction, lower loneliness, higher goal attainment, better subjective health and fewer functional limitations (*p*s ≤ 0.008)	*M* = 84, *SD* = 3, *R* = 80–93	64%
White et al., 2010	93	RCT	Computer-variety	A trend toward decreased loneliness (*p* < 0.52) and depression (*p* < 0.39) in intervention group compared to controls at post testing but not significant	Exp: *M* = 71, *SD* = 12, *R* = N/A Control: *M* = 72, *SD* = 11, *R* = N/A	Exp: 71% control: 82%
Quittschalle et al., 2020	999	Cross-sectional survey	Computer-variety (health-related)	ICT use associated with better quality of life (*p* = 0.006), lower levels of depressive symptoms (*p* = 0.04) and wider social network size (*p* = 0.01)	*M* = 80.49, *SD* = 4.69, *R* = 75–99	59.10%
Teo et al., 2019	1,424	Longitudinal survey	Computer-variety	Users of video chat had lower depressive symptoms compared to those who did not use video chat (*p* < 0.001); no association of depression score with social media, email or instant messaging use	*M* = 64.8, *SD* = 0.37, *R* = N/A	53%
Nimrod, 2012	218	Cross-sectional survey	Computer-variety	Participating in online communities provided joyfulness, stimulation and companionship. Online anonymity makes self-disclosure easier	*M* = 64.7, *SD* = N/A, *R* = 55–75	56%
Lelkes, 2013	11,000	Cross-sectional survey	Computer-general ICT	Those who suffer from loneliness or lack of social meetings, did not appear to benefit from internet use. Social isolation was lower among internet users. Positive association between regular internet use and self-reported life satisfaction	Age = 65+	N/A
Kim et al., 2016	6,476	Cross-sectional survey	Computer-general ICT	ICT use associated with increased likelihood of women visiting with family or friends (OR = 1.6, *p* = 0.002) and going out for enjoyment (OR = 1.3, *p* = 0.018). Association exists for men but only for going out for enjoyment (OR = 1.4, *p* = 0.036)	Age = 65+	56%
Lyu and Sun, 2020	7,193	Cross-sectional survey	Computer-general ICT	Social capital plays a mediating role in the relationship between Internet use and self-rated health among the older adults	*M* = N/A, *SD* = N/A, *R* = 60–95	48.99%
Tsai et al., 2010	57	quasi-experimental	Video-Skype/Windows Live Messenger	Video conference intervention: Experimental group had significantly better emotional (*p* < 0.01) and appraisal (*p* < 0.01) social support and loneliness (*p* = 0.02, *p* = 0.03) scores 1 week and 3 months after baseline compared to those in the control group. Depressive scores significantly (*p* = 0.02) lower at 3 months for experimental as compared to control group	Exp: *M* = 74.42, *SD* = 10.18, *R* = N/A control: *M* = 78.48, *SD* = 6.75, *R* = N/A	Exp: 58.3%, control: 57.6%
Jimison et al., 2013	9	Case series	Skype and health coach	Participants reported that Skype facilitated communication with remote family members and assisted in developing fast friendships that extended to additional social activities	*M* = 73.8, *SD* = 6.7 *R* = 76–92	89%
Tsai and Tsai, 2011	90	quasi-experimental	Video-Skype/Windows Live Messenger	After 3 months of videoconferencing with family the experimental group had higher changes in appraisal and emotional social support (*p* < 0.001). Experimental group had lower mean changes in loneliness (*p* < 0.001) and depressive scores (*p* < 0.001) evident at 3, 6, 12 months compared to baseline.	Exp: *M* = 73.82, *SD* = 11.19, *R* = N/A control: *M* = 79.26, *SD* = 11.19, *R* = N/A	Exp: 55%, control: 60%
Nam, 2019	1,132	Cross-sectional survey	Social media	Social media directly and indirectly (*via* perceived social support) influenced quality of life; men use social media for social support more than women	Age: 65+	54.90%
Aarts et al., 2015	626	Cross-sectional survey	Social media- social network sites	Usage unrelated to emotional/social loneliness or mental health	*M* = 66.94, *SD* = 5.99, *R* = N/A, 60 +	50.50%
Ivan and Hebblethwaite, 2016	13	Case series	Social media-Facebook	Video chat preferred over Facebook to build relationships with grandchildren	*M* = N/A, *SD* = N/A, *R* = 60–80	100%
Larsson et al., 2013	5	Case series	Social media	Social media use identified as complimentary to daily activities, not replacement; provided a way to be a part of grandchildren’s lives in a new way, increased knowledge about society, included and improved conversation. Participants worried about managing appearance on internet and privacy	*M* = N/A, *SD* = N/A, *R* = 65–85	60%
Bell et al., 2013	142	Cross-sectional survey	Social media (Facebook)	Social media users scored higher on social satisfaction and ICT confidence than non-users; no relationship with loneliness	*M* = 72, *SD* = 11, *R* = 52–92	66.90%
Pimentel et al., 2016	31	Case series	Smart phone-communication and social media apps	Participants reported that training allowed for greater socialization with family, friends and between colleagues of the course and increased independence and autonomy	Beginner class: *M* = 67, *SD* = 3.2, *R* = 58–77 advanced class: *M* = 66.8, *SD* = 3.3, *R* = 62–74	Beginner: 77.7% advanced: 61.5%
Pecino et al., 2012	165	Cross-sectional- survey	Smart phone-general	Having a mobile phone helped preserve friendships and increased independence, but not a means of social expansion	Mean = 62, *SD* = 5.46, *R* = N/A	66.7%

Exp = experimental, *M* = mean, *SD* = standard deviation, *R* = range, *N* = sample size.

### 2.5. Step 5: Summarizing and answering research questions

Once the data were charted, we then applied our research questions to the data. Specifically, we examined the types of interventions (e.g., what device or procedure), study and participant characteristics (study setting, demographics) and the reported impact or associations with loneliness (qualitative, quantitative, and inconsistencies) and social connectedness to create a narrative summary of the study objectives and findings. This allowed us to determine the relation among ICTs and older adults’ wellbeing.

## 3. Results

This review consists of the results of our examination of the characteristics and conclusions obtained from cross-sectional surveys and interviews/focus groups, quasi-experimental designs, randomized controlled trials, case series, and cohort studies.

Interventions are defined as studies where older adults were interacting or training with an ICT device either alone or in groups over a designated period of time, followed by providing feedback on how the interactions or training impacted their wellbeing *via* questionnaires or interviews. Of the case series, 16 out of 19 were longitudinal interventions that involved studying 1 group of older adults. The other non-intervention studies involved collecting user preferences at one point in time (i.e., scenario engagement) (Wherton and Prendergast, 2009; Papa et al., 2016) or conducting interviews with a single group of older adults on their ICT use (Ivan and Hebblethwaite, 2016). The cohort observational studies involved comparing two or more groups and were cross-sectional in nature. For example, (Melenhorst et al., 2016) recruited 24 e-mail users and 24 non-users to participate in a focus group to discuss communication scenarios, while (Tsai et al., 2015) compared prototype scenario engagement among the young-old, old-old and oldest-old participants. See Table 1 for a complete description of the characteristics of the 54 reviewed publications.

This review revealed that ICT devices were enjoyable (Straka and Clark, 2000; Nap et al., 2009; Gajadhar et al., 2010; Agmon et al., 2011; Nimrod, 2012; Chi et al., 2017); and using ICTs was associated with reduced social anxiety (Xu et al., 2016), higher wellbeing/life satisfaction (Shapira et al., 2007; Bell et al., 2013; Lelkes, 2013; Ballesteros et al., 2014; Sims et al., 2017; Nam, 2019; Quittschalle et al., 2020), improved self-efficacy (Straka and Clark, 2000; Woodward et al., 2010; Bell et al., 2013; Mitzner et al., 2019), greater self-esteem (Billipp, 2001; Bobillier Chaumon et al., 2013), and higher autonomy (Khvorostianov et al., 2011; Pecino et al., 2012; Pimentel et al., 2016; Pak et al., 2020).

There were associations between using ICTs and reduced loneliness (Shapira et al., 2007; Ballantyne et al., 2010; Tsai et al., 2010; White et al., 2010, 2016; Tsai and Tsai, 2011; Blažun et al., 2012; Cotten et al., 2013; Sims et al., 2017; Czaja et al., 2018) and social isolation (Lelkes, 2013) and depression (Shapira et al., 2007; Tsai et al., 2010; Tsai and Tsai, 2011; White et al., 2016; Czaja et al., 2018; Nam, 2019; Teo et al., 2019; Quittschalle et al., 2020). However, one study found no connection among ICTs loneliness or mental health (Aarts et al., 2015).

Generally, ICTs fostered social connections with family, friends and new acquaintances (Straka and Clark, 2000; Pfeil et al., 2009; Ballantyne et al., 2010; Khvorostianov et al., 2011; Blažun et al., 2012; Cornejo et al., 2012; Garattini et al., 2012; Nimrod, 2012; Pecino et al., 2012; Tsai et al., 2012, 2015; Winstead et al., 2012; Cotten et al., 2013; Jimison et al., 2013; Larsson et al., 2013; Arthanat et al., 2014; Huang and Hsu, 2014; Osmanovic and Pecchioni, 2015; Waycott et al., 2015; Delello and McWhorter, 2016; Ivan and Hebblethwaite, 2016; Melenhorst et al., 2016; Papa et al., 2016; Pimentel et al., 2016; Xu et al., 2016; Sims et al., 2017; Barbosa Neves et al., 2018; Czaja et al., 2018; Nimrod and Ivan, 2019; Zaine et al., 2019; Lyu and Sun, 2020). ICT use was associated with greater emotional and/or social support (Tsai et al., 2010; Woodward et al., 2010; Tsai and Tsai, 2011; Czaja et al., 2018) and social engagement (going out and hobbies) (Kim et al., 2016). They also increased leisure activities (Khvorostianov et al., 2011; Arthanat et al., 2014; Nimrod and Ivan, 2019).

Other articles reported on concerns older adults have with ICTs (e.g., managing appearance, privacy) (Nimrod, 2012; Larsson et al., 2013; Chi et al., 2017), as well as on older adult’s perceived usefulness of ICT devices, and emphasized the importance of including older adults in ICT designs/interventions (Wherton and Prendergast, 2009; Papa et al., 2016).

## 4. Discussion

The goal of this review is to investigate the relation between modalities of using ICTs and loneliness and social connectedness among older adults. Our examination of the literature suggests that the introduction of ICTs may improve the wellbeing of older adults in three major ways:

First, in the majority of studies reviewed here, evidence indicates that ICTs are associated with a reduction in loneliness, and depression. Second, ICTs promoted social connectedness by facilitating conversations. Third, we identified a series of other factors that contribute to the above-mentioned associations including, training, self-efficacy, self-esteem, autonomy, and the design/features, or affordances, of such devices. In the following sections, we discuss specifics of research studies reviewed, to provide a finer grained picture of the contextual specifics of studies that offer evidence to support our conclusions.

### 4.1. ICT use is associated with decreased loneliness, and depression and increased wellbeing

Findings from the scoping review allowed us to examine the specific ICT devices that were associated with reductions in loneliness and depression along with improvements in wellbeing. Interventions involving video conferencing (Tsai et al., 2010; Tsai and Tsai, 2011), web-based computer training (i.e., internet and social-networking websites) (Shapira et al., 2007; White et al., 2010, 2016; Blažun et al., 2012; Cotten et al., 2013) and two prototypes (Ballantyne et al., 2010; Czaja et al., 2018) were associated with decreased loneliness. Video conferencing provided older nursing home residents with emotional and appraisal social support (i.e., information that is useful for self-evaluation), and users experienced reduced depressive symptoms at three (Tsai et al., 2010; Tsai and Tsai, 2011) six and 12 months (Tsai and Tsai, 2011). Similarly, users of video chat had approximately half the probability of developing depressive symptoms, compared to non-users or those who used only email (Teo et al., 2019). Since video chat provides the richest media for mimicking in-person contact, it has a fundamental benefit for increasing social and emotional connectedness, in turn reducing feelings of loneliness (Tsai and Tsai, 2011; Teo et al., 2019; Ibarra et al., 2020). Such findings highlight the importance of creating ICT opportunities that are more like interpersonal exchanges to improve older adults’ wellbeing.

A quasi-experimental study noted older adults in their computer training course demonstrated decreased depression as compared to controls (Shapira et al., 2007). Cross-sectional and longitudinal survey results indicate ICT was associated with lower depression (Teo et al., 2019; Quittschalle et al., 2020). RCT designs have provided mixed findings on the effects of ICT training on loneliness and depression. An RCT prototype intervention completed individually by participants and provided easy access to resources and information sources and opportunities for engagement and communication (i.e., PRISM) was negatively correlated with depression scores (Mitzner et al., 2019). After conducting this RCT with 300 older adults, the group using PRISM, demonstrated greater perceived social support, and decreased loneliness at 6 months, but group differences disappeared at 12 months (Czaja et al., 2018). Another 12-month prototype intervention completed individually showed increases in wellbeing, but not in depression or activities of daily living (Ballesteros et al., 2014). RCTs involving computer training (e.g., learning computer basics) provide inconsistent findings. For example, 2 weeks of group computer training resulted in a trend toward decreased depression (White et al., 2010). Another 6-month group computer training intervention found greater self-efficacy and quality of life among the computer training group as compared to control but no differences in loneliness or depression (Woodward et al., 2010). A 3-month computer training study found weekly computer training with a nurse or significant other improved self-esteem compared to the control group who received only weekly nurse visits but no computer training. Additionally, those who trained with the nurse also demonstrated reduced depression (Billipp, 2001). Findings from RCTs highlight that the effectiveness of ICT interventions may be more closely related to who is conducting the training, rather than the training duration. Group versus individual based training formats are likely to affect training efficacy (Ibarra et al., 2020; Balki et al., 2022). These factors are discussed in more depth in Section 4.3.1.

Using web-based ICTs and using them frequently was associated with greater wellbeing compared to non-web-based ICTs or not using them at all. In a large-scale qualitative study of more than 1,600 older adults in Germany, individuals using web-based ICTs reported lower levels of loneliness compared to users of non-web ICT (e.g., TV) and non-users (Pak et al., 2020). Similarly, internet users expressed having greater and more social support networks than non-users, and users reported better self-rated health-related quality of life, fewer depressive symptoms, fewer chronic medical conditions and less feelings of loneliness (Quittschalle et al., 2020). Regular internet use was also associated with a lower chance of being socially isolated among older adults aged 65 and over even after controlling for personal characteristics such as income, marital status, gender, and health conditions (Lelkes, 2013). However, in another study, a higher frequency of online engagement was associated with lower levels of loneliness but not with lower levels of perceived social isolation among older adults in assisted and independent living communities. This finding suggests that *in person contact* rather than *online contact* may impact perceptions of social isolation (or social inclusion) (Cotten et al., 2013). Social networking websites seem to be related to temporary feelings of loneliness (i.e., loneliness which is experienced at a particular time of day or time of life) because of the flexibility of online communication, which can be used at any time of the day (Ballantyne et al., 2010; Nimrod, 2012). Facebook users were found to score higher on social satisfaction and confidence with technology as compared to non-users, but no differences in feelings of loneliness existed (Bell et al., 2013). Similarly, the greater the use of social media, the better the perception of social support, which increases participants’ quality-of-life (Nam, 2019). Among socially isolated older adults, ICTs that involved a closed social networking tool, such as posting photographs and messages on shared display for friends helped to facilitate social engagement and enhanced relationships between older adults and care managers (Waycott et al., 2015).

### 4.2. The contexts in which ICTs appear to have been helpful

The benefits of ICTs seem to be tied to strengthening pre-existing and new social connections and, promoting leisure and the fostering of intergenerational connections.

#### 4.2.1. ICT use strengthened pre-existing and new social connections

Multiple studies demonstrate that family and friends are important motivations for participating in and/or learning about ICT use (Ballantyne et al., 2010; Anderson-Hanley et al., 2011; Arthanat et al., 2014; Tsai et al., 2015; Barbosa Neves et al., 2018). Activities involving family connections were reported as being performed more frequently than those involving general social connection, leisure, health management, shopping, finances (Arthanat et al., 2014) or even obtaining new information (Sims et al., 2017). In turn, research indicates ICTs helped strengthen communication between older adults and their family and friends (Straka and Clark, 2000; Pfeil et al., 2009; Ballantyne et al., 2010; Khvorostianov et al., 2011; Pecino et al., 2012; Cotten et al., 2013; Jimison et al., 2013; Larsson et al., 2013; Huang and Hsu, 2014; Tsai et al., 2015; Delello and McWhorter, 2016; Ivan and Hebblethwaite, 2016; Melenhorst et al., 2016; Papa et al., 2016; Pimentel et al., 2016; Barbosa Neves et al., 2017; Sims et al., 2017; Czaja et al., 2018; Zaine et al., 2019). ICTs commonly reinforced in-person meetings and complemented offline communication with family, friends and new acquaintances (Pfeil et al., 2009; Larsson et al., 2013; Lelkes, 2013). ICTs also helped older adults remain in frequent contact with distant relatives (Khvorostianov et al., 2011; Melenhorst et al., 2016). Using ICTs to strengthen connections with others in turn is shown to potentially improve older adults’ sense of wellbeing by reducing loneliness and improving self-rated health (Cotten et al., 2013; Lyu and Sun, 2020).

We further investigated the types of ICTs used to facilitate social connections. For example, an ICT intervention that incorporated email, internet access, and online classrooms for social interactions made it easier to communicate with family and friends compared to a non-ICT intervention that included receiving a list of family/friends and other participant contacts to call (Czaja et al., 2018). Email frequently was used for exchanging light talk with friends and relatives (Pfeil et al., 2009). For in-depth conversations involving close personal relationships, one study identified that telephone or video chat was preferred over email or Facebook (Ivan and Hebblethwaite, 2016). Mobile phones were found to facilitate the provision of social support but they were not used extensively to maintain or enlarge older adults’ social networks (Pecino et al., 2012). Video conferencing increased older adults’ number of social contacts, and total online communication time with family and friends (Jimison et al., 2013). The use of an iPad-based communication app facilitated social connectedness in participants with geographically distant relatives (Barbosa Neves et al., 2017) and increased half the participants’ (6/12) communication frequency with social ties. However, the use of this application did not necessarily make relationships more meaningful as it was often used for brief contact or follow-ups. Other ICT prototypes such as ‘Media Parcels’ (sending and receiving pictures, audio clips, videos from other participants with the help of a facilitator to deliver the content) promoted social connection (Zaine et al., 2019).

Other ICT prototypes, not readily available to the public, hold promise. For example, one study reported the results of the use of a digital pet avatar with a voice activated cat or dog that was made available for 24/7 interaction through a live operator. Participants claimed that the device enhanced social interactions with other people as users could talk about their pet to friends and family (Chi et al., 2017). Another home-based communication system prototype encouraged peer-to-peer social engagement and offered older adults the chance to meet new people and promoted social connection online and offline (Garattini et al., 2012). Unfortunately, assessments of loneliness before and after the technology deployment were not considered.

Internet Communication and Technologies demonstrated to benefit relationships with others by reinforcing in-person meetings and internet contacts, however, they were predominantly seen as complementary, rather than supplementing in-person contacts (Lelkes, 2013). Social activities *via* social websites (i.e., Facebook, blogs, skype, MSN, 60 plus, and Stayfriends) and email were found to facilitate conversation with family, friends, and newly found acquaintances, and complemented both offline communication (Pfeil et al., 2009) and daily activities, rather than replacing them (Larsson et al., 2013). Using ICTs to connect with family and friends were found to be associated with greater life satisfaction, lower loneliness, and higher goal attainment (Sims et al., 2017). Connecting with family and meeting new people *via* internet was associated with lower levels of loneliness in residents of assisted and independent living communities (Cotten et al., 2013). Email and internet were beneficial for keeping in frequent contact with distant relatives over large geographic distances (Khvorostianov et al., 2011; Melenhorst et al., 2016). This was significantly associated with improved psychological wellbeing of older immigrants (Khvorostianov et al., 2011). However, it has been suggested that the internet is better at strengthening existing connections, rather than establishing new relationships (Cotten et al., 2013).

#### 4.2.2. ICT use for leisure and the fostering of intergenerational connections

Internet Communication and Technologies were found to enhance leisure experiences when online activities were both meaningful and enjoyable (Nap et al., 2009; Nimrod and Ivan, 2019). Some leisure environments fostered connections between older adults and younger generations (Agmon et al., 2011; Tsai and Tsai, 2011; Bobillier Chaumon et al., 2013; Larsson et al., 2013; Arthanat et al., 2014; Osmanovic and Pecchioni, 2015; Xu et al., 2016). Playing digital games was frequently encountered in the qualitative ICT literature emanating from leisure studies, where they were described as enjoyable, relaxing, and providing opportunities for skill enhancement (Nap et al., 2009; Osmanovic and Pecchioni, 2015; Nimrod and Ivan, 2019). However, here it is important to distinguish between different cohorts within the older population: those described as the “young-old” reported having a more favorable attitude than the “old-old” for using multimedia sharing and interactive games (Tsai et al., 2012; Xu et al., 2016). Playing games that were challenging and could enhance one’s skill set seems to motivate older adults to play digital games. However, negative perceptions of digital gaming exist within this population, including a fear of failure, as multiplayer games were competitive and revealed one’s skill set. For these reasons, the literature indicates that older adults prefer single player games (Nap et al., 2009) or cooperative game play (Osmanovic and Pecchioni, 2015; Xu et al., 2016).

Co-playing, in several studies, may increase feelings of connectedness between players and improve their engagement (Gajadhar et al., 2010; Xu et al., 2016), as highly interactive games simulated interpersonal conversation (Xu et al., 2016). These conclusions were supported in a survey of 124 adults, which concluded that older adults enjoy solo play on casual computer games for leisure and personal challenge, and social play for connection especially intergenerationally rather than competition (De Schutter, 2010; Khalili-Mahani et al., 2020). However, in an exergaming study by Xu et al. (2016), the age of the co-player was found to have differential effects on the “young-old” and “old-old” cohorts’ psychosocial wellbeing. A young-old cohort saw improvements in sociability including an interest in being around or socializing with others and a decline in social anxiousness after playing with adolescents. Players who were categorized as “old-old” only reported improved sociability from playing with another older adult. In both cases, it was not the intervention but the togetherness that produced positive effects. Both groups showed decreased loneliness scores after game play (Xu et al., 2016).

Other leisure-based ICTs, such as engaging with the websites of cultural institutions like museums or using Google Earth for sight-seeing, facilitated older adults’ participation in these types of social activities and virtual environments that would have been inaccessible due to social or physical barriers. However, from the user’s perspective, leisure-based ICTs have a paradoxical relation with time. While the internet made it easier to partake in some leisure activities, such as finding and listening to music, it was also described in the negative context as something that absorbed or wasted time (Nimrod and Ivan, 2019). The importance of considering the affordances of the device to enhance wellbeing through fostering intergenerational relationships are supported by a 2019 field study by Marston et al. (2019), a social media study by Khalili-Mahani et al. (2021), and a recent scoping review by Ibarra et al. (2020).

Taken together, it is clear that ICTs foster social connections in two primary ways: (1) ICT use strengthens pre-existing and new relationships, and (2) ICTs are used for leisure, which fosters social connectivity and intergenerational connections.

### 4.3. Additional factors contributing to the association between ICT use and wellbeing

A series of factors positively contribute to the association between ICT use and older adults’ wellbeing, which include ICT training, ownership and design/features. These factors have the ability to improve social connections, autonomy and self-efficacy, and reduce loneliness, among other aspects of quality of life.

#### 4.3.1. The association between ICT training and loneliness and social connectedness

When determining the factors that positively contribute toward the association between ICT use and older adults’ wellbeing, ICT training appears to play a critical role. Previous studies have identified that older adults who have taken computer training to learn basic computer skills (turning computers on, internet searching, and e-services) have reported reduced levels of loneliness and an increase in their social networks. For example, among older adults living in institutional care homes who face barriers to socializing because of mobility or health problems, learning ICT skills has shown to increase their social networks and reduce levels of loneliness (Blažun et al., 2012). Similarly, older adults provided with ICT training to communicate with family and finding information exhibited lower levels of loneliness. These findings were apparent in both assisted and independent living communities (Cotten et al., 2013). Such findings highlight that ICT training can have a positive contribution toward participants’ social relationships making it easier to connect with family and meet new people.

Another form of ICT training can occur in groups, which creates a community of learners who may assist one another with their technology skills (Delello and McWhorter, 2016). Group ICT training formats (basic lessons, web searching, etc.) such as those by Winstead et al. (2012) and White et al. (2010, 2016) involve an instructor and/or assistant who help multiple participants. Researchers have indicated that training programs are associated with increased social networking interactions (Straka and Clark, 2000; Winstead et al., 2012), decreased loneliness (Shapira et al., 2007; White et al., 2010), reduced feelings of depression, improved satisfaction and quality of life (Shapira et al., 2007; Woodward et al., 2010), improved perceived social support (Woodward et al., 2010), and emotional state (Silva et al., 2018). However, given that ICT group training are often formed around the shared ICT experience makes it difficult to determine how much of the positive psychological effect is due to ICT use or to the group social interactions that occur during these sessions (Straka and Clark, 2000; White et al., 2010). Potential benefits from group ICT training may be related to the support provided by facilitators or family members (Billipp, 2001). Additionally, others suggest that the novelty of learning to use ICTs play a role in promoting wellbeing (Blažun et al., 2012). For example, after 6 months of ICT training there was a significant improvements in perceived social support, wellbeing and decreased feelings of loneliness. Yet these positive effects wore off at 12 months. It may be the case that the novelty of the ICT intervention disappeared after 12 months (Czaja et al., 2018). Regardless, both independent and group-based ICT training formats appear to be associated with older adults’ wellbeing, at least in the short-term.

#### 4.3.2. ICTs are positively related to self-efficacy, self-esteem, autonomy, and independence

In addition to the social benefits of ICT training, learning how to use ICTs can contribute to improvements in ICT self-efficacy (belief in ability to use ICTS), self-esteem and autonomy, by providing users with the opportunity to engage with society. More specifically, after introducing ICTs to older adults, many reported improved independence and autonomy (Straka and Clark, 2000; White et al., 2010; Bobillier Chaumon et al., 2013; Pimentel et al., 2016); self-esteem and ICT self-efficacy (Billipp, 2001; Woodward et al., 2010; Bobillier Chaumon et al., 2013; Tsai et al., 2015; Pimentel et al., 2016). It is of great importance that older adults believe in their capacity to use ICTs, a hallmark of self-efficacy, as it is a predictor for both mid-term and long-term ICT adoption and use (Czaja et al., 2006; Mitzner et al., 2019; Jokisch et al., 2021). One study identified the significance of age in relation to ICT and self-efficacy. Tsai et al. (2012) found that the oldest-old participants had a lower sense of self-efficacy when it came to multimedia sharing and interactive gaming (Tsai et al., 2012). Additionally, the type of ICT device used may strengthen feelings of independence. For example, using a mobile phone increased feelings of security and independence among older adults (Pecino et al., 2012). Additionally, survey results from older adults living in private homes and institutional settings found that in comparison to non-users or users of non-web ICTS, those who used web-based ICTs reported higher levels of autonomy (capacity to decide how to act and being accountable for actions) and lower levels of anomie (feelings related to coping with the current social standards, compatibility of one’s own values to those in society and orienting oneself in fast changing society) (Pak et al., 2020). The benefits of learning how to use ICTs gave older adults a sense of belonging (Shapira et al., 2007) and an increased awareness of world events (Winstead et al., 2012; Tsai et al., 2015). This contributed to enhancing older adults’ ICT competency (Bobillier Chaumon et al., 2013; Tsai et al., 2015), which provided them more opportunity to have conversations with family and friends about current events (Bobillier Chaumon et al., 2013; Larsson et al., 2013).

#### 4.3.3. ICT design and features

Given there are many prototype studies emerging which examine the relation between ICTs and wellbeing, it is important to consider which design features or affordances of the device promote rather obstruct ICT adoption and engagement. We identified a number of prototype interventions and ICT engagement scenarios, which focused on the user interface and user experience (UI/UX) (Wherton and Prendergast, 2009; Ballantyne et al., 2010; Cornejo et al., 2012; Garattini et al., 2012; Tsai et al., 2012; Jimison et al., 2013; Huang and Hsu, 2014; Waycott et al., 2015; Papa et al., 2016; Chi et al., 2017; Czaja et al., 2018; Zaine et al., 2019). Designing ICTs with attention to the physical and cognitive needs of older adults may increase their usage and facilitate efficacy in reducing loneliness. Several studies focused on improving UI/UX by adapting the ergonomics of the ICT systems from the perspective of older users to facilitate interactions and increase use. For example, the use of EasyReach (a TV social channel with social networking opportunities) was developed for social interaction with near and distanced friends and family. While users perceived it as a way to reduce feeling of loneliness, difficulties in learning to use the system interfered with older adults’ abilities to benefit from the device (Papa et al., 2016).

Managing appearance and privacy were two of the important affordances that older users identified as crucial to their desire to use ICTs (Larsson et al., 2013). Online communities that had features that allowed for anonymity and invisibility reduced social anxieties and afforded more confidence when talking to others and trying new things (Nimrod, 2012). Although ICT prototypes, such as pet avatars, were found to be beneficial in fostering social connectedness, some of their limitations included the absence of a face-to-face component, (Garattini et al., 2012) and concerns regarding the quality of the social interaction between participant and prototype (Chi et al., 2017). Older adults frequently reported concerns about the complexity of technology, as well as the security of private information, including identity theft (Pfeil et al., 2009; Wherton and Prendergast, 2009; Garattini et al., 2012).

Internet Communication and Technologies which featured open chats, were found to have caused frustration among users. For example, frequent system users became frustrated when they received no responses or delayed responses when sending messages or calls (Garattini et al., 2012). These frequent users were women and individuals showing indications of social loneliness which suggest gender (Garattini et al., 2012; Kim et al., 2016) and levels of loneliness (Garattini et al., 2012) are factors to consider when designing ICT features and interventions. As Wherton and Prendergast (2009) have shown, it is important to be mindful of the gender inequality gap and keep ICTs simple and consistent with the needs and requirements of users to promote ICT adoption by older adults.

### 4.4. Challenges and limitations

A common challenge in reviewing the literature on the association between ICT interventions, loneliness and social connectedness was the heterogeneity of methodologies, statistical reporting (i.e., effect sizes), range in sample sizes across studies, and the diversity in ICT devices. For example, this review includes RCTs, cross sectional designs, quasi experiments, case series and cohort studies. Sample sizes ranged from 1 to 11,000 and there were seven different ICT categories (i.e., digital games, iPad, prototypes, computer, video, social media, and smartphone). Within each category there was heterogeneity in the features of the devices and interventions A lack of methodological consistency contributes to result inconsistency.

The diversity in this data set prohibited us from making conclusive assertions about what factors explain or obscure the association between ICTs, loneliness, and social connectedness. For instance, potential benefits from group ICT training may be to a larger extent related to the support provided by facilitators or family members than the intervention itself (Billipp, 2001). Or, novelty may have played a part in improving wellbeing, by providing new opportunities to enhance leisure, communication, and social connectedness during the experiment, but it is not clear whether such effects would remain.

Given the literature searches were performed in 2020, we did not include articles published during the COVID-19 pandemic. However, our review provides a first step in mapping the ways in which ICTs are related to loneliness and social connectedness not affected by the pandemic. This is important as ICT use/intervention studies during the pandemic would have removed features of in-person social contact, which as demonstrated in our review, can affect the association among ICT use, loneliness, and social connectedness. Future researchers may find this article useful when comparing ICT interventions pre, during and post pandemic. Final limitations of our review are that it is specific to healthy older adults and excludes those with impairments or serious health conditions. Such conditions are prevalent with aging. Therefore, future reviews should strive to include them in their research. Additionally, we did not account for possible publication biases.

## 5. Conclusion

### 5.1. Summary of findings

Prior to this review it was unknown to what extent ICTs were associated with loneliness and social connectedness. The objective was to identify the dominant themes and findings in the literature surrounding this topic and to try and uncover what other factors may be contributing to these associations. We have provided readers with a way to map out ways in which these associations may or may not exist. For example, we conclude that the majority of studies surveyed demonstrate that ICT use among older adults is associated with reduced loneliness by supplementing existing social connections, by allowing for reconnection and formation of new relationships. ICTs that provide opportunities for leisure and learning are found to be enjoyable and foster intergenerational connections, which in turn has positive effects on psychosocial wellbeing. Learning and/or training to use ICTs has a positive relation to self-efficacy, self-esteem and autonomy, and independence. As such, our findings highlight the necessity of addressing the heterogeneity of older adults’ and their ICT preferences, motives, capabilities and concerns, and most importantly pre-existing social connections, to address the intertwined complexities among ICTs, loneliness, and wellbeing.

### 5.2. Implications for future studies

A strength of this scoping review is that it utilizes research from a range of multidisciplinary databases within the fields of Psychology, Sociology and Medicine. This extensive literature search provides an overview of the scholarly work in this field (more than 8,000 articles satisfying the initial search) and the limited number of empirical studies that satisfied our inclusion criteria (only 54 articles). The literature covered within this review encompasses both qualitative and quantitative methodologies. It is clear that a mixed methods approach provides a deep and nuanced perspective on the multiplicity of factors at play in the study of how older adults engage with ICTs and how given interventions improve wellbeing. At the same time, our review reveals the necessity of considering the context of ICT use, and the overall adoption of ICTs over the life course. Operational complexity of accounting for these variations in controlled trials underlines the difficulty of solely quantitative methodologies in establishing the health benefits of ICT interventions. Such a perspective goes beyond measuring the variations that arise from the biological factors associated with aging, as it takes seriously the psychological, social and cultural conditions that modulate the experiences of older adults in their homes or even the laboratories where some of these empirical experiments have taken place. Paying attention to these variations is a reminder of the range of motivations for the use of ICTs by older adults, as well acknowledging their preferences and their agency. Additionally, this review does not appraise the quality of evidence in terms of which ICT devices or interventions effectively reduce loneliness and improve wellbeing. As such, a systematic review focusing on this causal relation would be of great benefit to this field of research and those designing ICTs, but a greater number of RCTs is needed to perform this review. This review can help inform future researchers to consider the importance of implementing aspects of social connection, training format, leisure, easy to use design features and affordances into their ICT designs or interventions as a way to improve wellbeing. We have provided a starting point for future mediation and moderation analyses given we have identified several contributing factors to ICT and social construct associations.

## Author contributions

SH, BP, and CM identified a research question. BP and CM performed a literature search, selection, and review. BP, NK-M, and SH analyzed the data, extracted the themes, and wrote the manuscript. All authors provided feedback, reviewed the article, and approved the submitted version.
